# GNINA 1.0: molecular docking with deep learning

**DOI:** 10.1186/s13321-021-00522-2

**Published:** 2021-06-09

**Authors:** Andrew T. McNutt, Paul Francoeur, Rishal Aggarwal, Tomohide Masuda, Rocco Meli, Matthew Ragoza, Jocelyn Sunseri, David Ryan Koes

**Affiliations:** 1 Department of Computational and Systems Biology, University of Pittsburgh, Pittsburgh, PA USA; 2 Center for Computational Natural Sciences and Bioinformatics, International Institute of Information Technology, Hyderabad, 500 032 India; 3 Department of Biochemistry, University of Oxford, Oxford, United Kingdom

**Keywords:** Molecular docking, Deep learning, Structure-based drug design

## Abstract

Molecular docking computationally predicts the conformation of a small molecule when binding to a receptor. Scoring functions are a vital piece of any molecular docking pipeline as they determine the fitness of sampled poses. Here we describe and evaluate the 1.0 release of the Gnina docking software, which utilizes an ensemble of convolutional neural networks (CNNs) as a scoring function. We also explore an array of parameter values for Gnina 1.0 to optimize docking performance and computational cost. Docking performance, as evaluated by the percentage of targets where the top pose is better than 2Å root mean square deviation (Top1), is compared to AutoDock Vina scoring when utilizing explicitly defined binding pockets or whole protein docking. Gnina, utilizing a CNN scoring function to rescore the output poses, outperforms AutoDock Vina scoring on redocking and cross-docking tasks when the binding pocket is defined (Top1 increases from 58% to 73% and from 27% to 37%, respectively) and when the whole protein defines the binding pocket (Top1 increases from 31% to 38% and from 12% to 16%, respectively). The derived ensemble of CNNs generalizes to unseen proteins and ligands and produces scores that correlate well with the root mean square deviation to the known binding pose. We provide the 1.0 version of Gnina under an open source license for use as a molecular docking tool at https://github.com/gnina/gnina.

**Supplementary Information:**

The online version contains supplementary material available at 10.1186/s13321-021-00522-2.

## Introduction

Molecular docking is a computational procedure in which the non-covalent bonding of molecules, e.g. a protein receptor and a ligand, is predicted. This prediction outputs the conformation and, usually, the binding affinity of the small molecule in its predicted minimal energy state and is used to virtually screen large libraries of compounds [1–3]. Docking is composed of two main steps: sampling and scoring. Sampling refers to an extensive search of the conformational space of the molecules being docked. This conformational space is vast, due in part to both the receptor and ligand being flexible allowing for each molecule to adjust its shape due to the influence of the other. In order to constrain this large conformational space, the receptor is typically kept rigid. The other vital piece of molecular docking is the scoring function. Every sampled pose is evaluated by the scoring function for its fitness. The fitness determines the conformations that are retained from sampling and is used to rank the retained poses in the order of their likelihood of being correct. The final output of docking is a set of ranked poses of the docked molecule.

Determination of the correct binding pose of a small molecule is a prerequisite for determining its binding affinity and affords the opportunity to utilize the pose for lead optimization. Correct evaluation of binding affinity is critical for downstream tasks such as virtual screening or for determining if a compound is important for more experimental analysis. Molecular docking must compute a pose and a binding affinity quickly for it to beneficial when millions of ligands are being queried in a drug discovery pipeline [1]. Sampling the entire conformational space of a molecule is computationally demanding; therefore, we compromise on the speed and accuracy of docking to provide poses that are close to native while not requiring the full search of conformational space. This compromise requires docking software to focus on the accuracy and ranking power of the scoring function to highlight low energy conformations and reduce extraneous sampling.

Scoring functions provide a mapping from the conformational space of the ligand and receptor to the set of real numbers so that poses may be ranked. Typically, scoring functions are grouped into three categories; knowledge based, physics based, and empirical [1]. Knowledge based scoring functions leverage the statistics from a set of structural binding data. A number of geometric properties are computed from structures of protein-ligand complexes, such as atom-atom pairwise contacts. The calculated frequencies can be used in a method such as potential of mean force (PMF), which creates a potential based on the Boltzmann distribution of the properties, to calculate the score of a pose [4, 5]. Knowledge based scoring functions can be biased by features present in their training sets though calculations of scores are quick at test time [1]. They require a large database of known structures and can be difficult to interpret when trying to understand a score [6]. Physics based scoring functions, often referred to as force fields, utilize physically derived energetics of interactions to compute scores. The final score is a summation of energy terms such as Coulombic and Van der Waals forces [7]. Accuracy of physics based scoring functions are limited by their complexity and the assumptions we place on the fundamental forces dictating interactions between molecules, though understanding of these forces is continually increasing [8].

Empirical scoring functions address the limitations of physics based scoring functions by using a combination of manually selected energy terms. Rather than giving each energy term identical weighting, the weights of each term are determined via a fit to experimental data. A large proportion of docking software use empirical scoring functions, including X-Score, AutoDock Vina, and ChemScore [9–11]. Unlike knowledge based scoring functions, empirical and physics based scoring functions may be easily interpreted to determine the contributing factors of a given score since each energy term can be individually queried. Fitting empirical scoring function requires a plethora of experimental structural data and prevents the combination of terms from separately trained scoring functions. The three categories of scoring functions are limited to features extracted from structural information and often assume there is a linear relationship between the features and the binding affinity. AutoDock Vina (called “Vina”) utilizes an empirical scoring function explicitly tuned to structural data [10]. The Vina scoring function is a weighted sum of atomic interactions. Steric, hydrophobic, and hydrogen bonding interactions are calculated and are adjusted by the number of rotatable bonds to account for entropic penalties. The weights of the terms were computed via a non-linear fit to structural data. Nguyen et al. [12] show that Vina can more accurately predict the binding pose than its predecessor, AutoDock 4 [13]. Vina demonstrates the power of modelling non-linear relationships with its increased docking performance. Therefore, we search for alternative scoring functions that are able to model non-linear relationships between inputs.

Machine learning (ML) represents another growing class of scoring functions [8]. ML algorithms learn arbitrary relationships between observations and outputs while classical scoring functions assume a specific functional form [14]. There has been considerable progress in other biomedical fields with the utilization of ML models [15]. However, machine learning algorithms require a large amount of data to properly generalize to unseen information. The last 20 years has seen a noteworthy increase in the quantity of available protein structures [16]. A plethora of databases annotate structural data with experimental binding affinity data, including PDBbind and BindingDB [17–19]. This information has been utilized to leverage machine learning algorithms as scoring functions. A number of traditional ML approaches have been used as scoring functions, including random forests (RF-Score [20] and SFCScore [21]), support vector machines (SVR-Score [22], ID-Score [23], SVR-EP [24]), artificial neural networks (NNscore [25] and BsN-Score [26]), and gradient boosted decision trees (BT-dock [27] and ESPH T-Bind [28]). These ML methods have been able to match or exceed existing traditional scoring functions. ML methods allow a more robust fit to training data, but are limited to features manually extracted from structural data.

Deep learning (DL) methods allow direct inference of features from inputs. They learn a representation of the inputs via layers of simple, non-linear models which transform the representation to higher abstractions to learn complex functions [29]. DL methods have demonstrated success in a variety of fields, such as computer vision and natural language processing [30, 31]. In recent years, there has been significant progress with DL methods in the drug discovery field with many models employing a convolutional framework. Convolutional neural networks (CNN) leverage convolutions to infer features directly from input tensors, usually images. CNNs have shown potential in virtual screening (AtomNet [32], DeepVS [33], Ragoza et al. [34]) and binding affinity prediction (PotentialNet [35], $K_{DEEP}$ [36], Pafnucy [37]). A number of methods have been proposed to capitalize on the power of DL scoring functions. MedusaNet uses a CNN within the docking pipeline to guide the sampling of the base docking method [38]. The base docking method, Medusa, provides a variety of ligand poses. The CNN evaluates the 3D coordinate representation of the poses to determine if a pose should be retained. Nguyen et al. [39] describe a generative adversarial network (GAN) for pose prediction. Their network utilizes an encoder with low-dimensional mathematical representations of the protein-ligand complex and a decoder utilizing convolutional layers to generate and rank ligand poses for the D3R grand challenge. Masuda et al. [40] use a receptor structure as the prior to their GAN to sample novel ligands appropriate to the identified binding site.

Previous work has largely evaluated deep learning protein-ligand scoring on already generated poses. In this work, we describe and comprehensively evaluate version 1.0 of the Gnina molecular docking software, a fork of Smina [41] and AutoDock Vina [10] that supports CNN scoring as an integral part of the docking workflow. Gnina is evaluated here for its ability to properly score and rank binding poses for protein-ligand complexes. We describe how the default settings which balance docking accuracy and runtime were determined, including the selection of a default ensemble of CNN models. Performance of Gnina is evaluated for the redocking, cross-docking, flexible docking, and whole protein docking tasks and is found to significantly outperform Smina/Vina in all cases.

## Methods

**Fig. 1:**
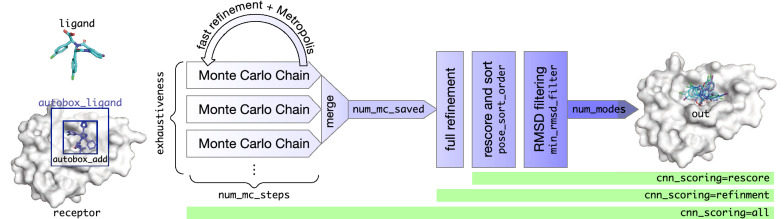
The Gnina sampling and scoring algorithm shown with relevant commandline parameters and the scope of CNN scoring

The docking pipeline of Gnina (Fig. 1) is described in detail, providing background for the derivation of default usage. A default CNN ensemble is selected for optimizing the docking performance and runtime of the docking pipeline. This ensemble is then used to investigate the different CNN scoring options available to the user, followed by a thorough investigation of the docking parameters. Finally, we examine both the generalizability and scoring power of Gnina.

### Molecular docking pipeline

**Fig. 2:**
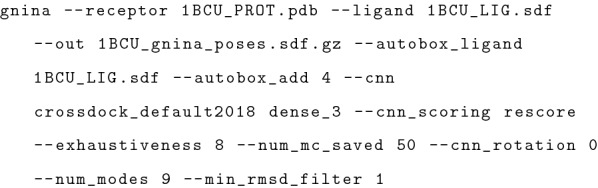
Example Gnina usage

Gnina is a fork of Smina [41] which is a fork of Vina[10]. The docking pipeline of Gnina utilizes the enhanced support for scoring enabled in Smina to allow the use of CNNs as scoring functions. In typical usage, Gnina is provided with a receptor structure, a ligand structure, and a specification for a binding site on the receptor (Fig. 2).

Open Babel [42, 43], a chemical toolbox allowing the reading and writing of over 100 chemical file formats, is used for parsing the inputs, allowing commonly used structural data formats (e.g. PDB, sdf, mol, etc.) as well as gzipped versions of such files to be used. The binding site can be specified as a Cartesian box or by providing a ligand file (autobox_ligand). When autobox_ligand is used to define the binding site, a rectangular prism is constructed using the minimum and maximum values for the *x*, *y*, and *z* coordinates of the ligand to which additional spacing (autobox_add) is added in every dimension (Figs. 1, 2). In Gnina, if any side of this auto-generated box is smaller than the longest distance between any two atoms in the ligand, then those sides are extended to that longest distance, ensuring that the ligand can rotate freely within the defined box without incurring an out-of-box penalty that is applied to all docked poses to constrain them to the specified binding site search space.

Next, the scoring functions are setup. Similar to Smina, if the user opts to not use the CNN scoring function for a part of the pipeline they can specify their own empirical scoring function to Gnina or choose one of the built-in scoring functions, i.e. Vina, Vinardo [44], etc. CNN scoring functions can be specified by providing model and/or weights files or by selecting a built-in model. The available built-in CNN models include crossdock_default2018, dense, general_default2018, redock_default2018, and default2017, each of which is trained using different training data and/or a different model architecture. Additionally, each model, except for default2017, has five variants that are trained on the same data and have the same architecture, but are initialized with a different random seed. For each CNN model type, we refer to the variant with the highest docking performance as the base model name, and the remaining variants are given sequential numbers (i.e. general_default2018, general_default2018_1, general_default2018_2, general_default2018_3, and general_default2018_4); the ensemble of these five variants is denoted with ‘_ensemble’ (i.e. general_default2018_ensemble). The architecture and training of these models are described elsewhere [34, 45]. CNN calculations are performed using the cuDNN accelerated Caffe deep learning framework [46]. These models are trained to predict both a pose score (a probability that the pose has a low root mean square deviation (RMSD) to the binding pose) and the binding affinity (pK). The pose score is used for all pose optimization tasks. The scoring function used in each step of the Gnina pipeline is determined by cnn_scoring (defined below), defaulting to only using the CNN scoring for the final resorting of ligand conformations and the empirical scoring function everywhere else in the pipeline.

The docking procedure uses Monte Carlo sampling to search the ligand conformational space. exhaustiveness (default 8) defines the number of Monte Carlo chains that are run for the ligand. Chains are run in parallel using up to cpu threads. The number of steps for the Monte Carlo chains are calculated based on the number of mobile atoms and the number of degrees of freedom within the ligand. This calculation can be ignored and the user may explicitly specify the number of steps for the Monte Carlo chains with num_mc_steps. Each step of a Monte Carlo chain mutates the ligand by randomly selecting one of the following operations: random translation, random rotation of the entire molecule, or randomly setting the torsional angles of the ligand. The Monte Carlo process selects random resetting of the torsional angles with a higher probability than the other mutations. After the mutation, an approximate energy minimization of the ligand is performed. If an empirical scoring function is specified for guiding sampling, this minimization is performed using a fast, grid-based approximation of that scoring function. A grid is pre-calculated for each ligand atom type and a single atom of that type is used to calculate values for each grid point. The full ligand is scored by interpolating values from the grid for each of its atoms and summing the result. If a CNN scoring function is specified, no such approximation is used since CNN scoring is not additive with respect to the individual atoms [47]. The score for the minimized conformation determines if it will be accepted using the Metropolis acceptance criterion. Each Monte Carlo chain retains its top scoring ligand conformations, and the number retained is user configurable with num_mc_saved (default 50).

Following the completion of Monte Carlo sampling, the saved conformations from each Monte Carlo chain are aggregated and the num_mc_saved top scoring conformations are retained for further analysis. The top scoring conformations are then refined. If an empirical scoring function is specified for refinement (cnn_scoring set to “none” or “rescore”), a functional formulation, rather than a grid approximation, of the scoring function is used to carry out refinement in order to get the locally optimal pose. Refinement shifts the ligand pose to a local energy minimum using the gradients of the scoring function. After the ligand pose has been refined, the final affinities and scores are calculated for the pose using the specified CNN models and/or the specified scoring function. Finally, the top scoring conformations are sorted by pose_sort_order (default “CNNscore”) and output with Open Babel in the user-specified format if an output file was provided.

The usage of the CNN models within the docking pipeline can be selected by the user using cnn_scoring (Fig. 1, 2). If “none” is selected for cnn_scoring, then the CNN models are not used at all in the docking pipeline, making the pipeline essentially identical to the Smina pipeline. The only differences are that Smina computes with double (64 bit) precision rather than the single (32 bit) precision used by Gnina and Smina does not include the autobox_extend parameter when creating the sampling box. When “rescoring” is selected (the default), the specified CNN models are not used until the final sorting of resultant ligand conformations after their refinement with the empirical scoring function. In this case, the specified CNN models are used to score each of the ligand conformations and the output ligand conformations are resorted based on the score calculated by the CNN model(s). The “refinement” option utilizes the CNN for the refinement of the ligand poses after they have been selected by the Monte Carlo chains and then sorts the refined ligand conformations by the CNN score for output. Finally, the “all” option utilizes the CNN for all aspects of the docking pipeline including the minimization within the Monte Carlo chains, the refinement after the Monte Carlo chains, and the sorting of the final output.

#### Flexible docking

Molecular docking is often performed using a rigid protein target and only samples the conformational space of the ligand, as described above. This is a good approximation when redocking to a receptor that does not undergo conformational change upon ligand binding, but it fails to accurately represent the biochemical aspects of the system when the protein undergoes significant structural changes upon binding [48]. Allowing the whole target to be flexible is too computationally expensive for docking; however, some protein flexibility can be accounted for by sampling the conformational space of side chains in the binding site [49] while leaving the backbone fixed. The local flexibility of the binding site could be especially beneficial for cross-docking, i.e. docking a ligand to a non-cognate receptor. Assuming a rigid receptor during cross-docking is not realistic when potentially very different ligands are docked to the same receptor.

Gnina allows the sampling of side chain, but not backbone, conformational space. More specifically, side chain torsion angles are sampled as part of the Monte Carlo search. Side chain flexibility can be specified manually or semi-automatically in several different ways:

- flexible side chains can be defined in a PDBQT file (flex parameter),
- they can be selected using a comma-separated list of residue identifiers (chain, residue number, and, optionally, insertion code; flexres parameter),
- or they can be selected based on their distance from a given ligand (flexdist and flexdist_ligand parameters).

The last option, used in this work, is similar to the autobox_ligand option described above. The flexdist parameter allows the specification of a threshold distance from the flexdist_ligand. If a residue has any side chain atoms that are within this distance of the specified ligand, then the entire residue side chain is marked as flexible.

Given the increased computational cost of sampling side chain conformations, two additional options are provided for flexible docking. flex_limit allows users to specify a hard upper bound for the number of flexible side chains. If flex_limit is exceeded for a particular system, docking does not run and Gnina terminates with a warning. This option is particularly useful to avoid possible bottlenecks in large virtual screening tasks. The less strict flex_max option allows users to prune the list of flexible side chains in order to make the calculation more manageable for large systems; if there are more than flex_max side chains identified as flexible, only the conformational space of the flex_max closest to flexdist_ligand is sampled during docking.

Flexible side chains are selected at the very beginning of the docking procedure and the selection is not updated during sampling. When using autobox_ligand to automatically define the search space as described above, flexible side chains are included in the calculation of the box bounds. The usage of CNN models for docking with flexible side chains can be selected by the user in the same way it is done for docking with a rigid receptor, as described above.

### Data

There are two primary ways to evaluate molecular docking: redocking a cognate ligand to its receptor and docking a ligand to a non-cognate receptor (cross-docking). In order to best evaluate the performance of Gnina for molecular docking, we evaluate its performance on both of these tasks. Redocking the cognate ligand demonstrates the sampling and scoring power of the molecular docking pipeline, as the RMSD from the crystal pose can be measured to exactly determine the accuracy of the produced poses. Analysis of redocking requires a set of high quality structures in which the native binding pose of the ligand has been solved. For this purpose we utilize the PDBbind refined set v.2019 [18]. The PDBbind database is a curated set of protein-ligand complexes containing both structural information and binding affinity. The PDBbind database is updated annually with new experimentally determined structures annotated with binding affinity data. The refined set is a subset of the entire PDBbind dataset that retains only the structures with resolution higher than 2.5 Å, high quality affinity measurements, and binary protein-ligand complexes. The 2019 release of the refined set contains 4,852 high quality crystal structures of native protein-ligand binding poses.

However, redocking is not the normal use case of a molecular docking pipeline. Docking will ordinarily be performed on proteins-ligand pairs that have no co-crystallized structure. Often a new ligand will be docked into a receptor whose co-crystallized ligand is a different molecule. Wierbowski et al. [50] recently published a dataset that provides a benchmark precisely for this task. This cross-docking dataset provides a meaningful method for the evaluation of the ligand RMSD from the known and predicted poses. A reference structure is selected for the protein, then the “known” binding pose of a ligand is defined by the ligand’s position when an alignment is performed between the reference structure and the ligand’s co-crystal receptor structure. The dataset is composed of 94 unique protein binding pockets and 4,399 unique ligands, with an average of 46 ligands per target.

Both of the datasets were filtered to ensure the protein-ligand structures can be parsed by Gnina. ProDy [51] was used to separate the complexes into protein and ligand files while removing any water or other extra crystallized molecules. Our goal is the binding pose prediction of a small molecule at its target site, therefore we utilized RDKit [52] to filter both the redocking and cross-docking datasets to include only ligands with molecular masses greater than 150 Da and less than 1000 Da. Any ligand that was not able to be parsed with RDKit was also removed. In addition, we visually inspected the ligands of the cross-docking dataset with Pymol [53] to ensure that each ligand for a given binding pocket overlapped. If a ligand was non-overlapping it was marked as problematic. Lastly, for the UROK pocket, 4ZKO was removed as the receptor did not align properly and the ligands from 4MNW and 4MNX were also marked as problematic due to their binding pose depending on a chain from the kinase being in a protein-protein complex rather than a holo structure as in the other members of the pocket. The final filtered datasets were composed of 4,260 and 820,280 protein-ligand pairs for the redocking (PDBbind [18]) and cross-docking (Wierbowski et al. [50]) datasets, respectively.

Due to the combinatorial size of the cross-docking dataset, more filtering was required to make computational time tractable. Each unique protein binding pocket can be used to group the protein-ligand complexes as all of the receptor structures in the group share a common pocket. These groupings allow the cross-docking dataset to be downsampled for each pocket. Each pocket was either kept in full, or reduced to a random sample of 100 receptor-ligand pairs, whichever was smaller. We then removed any of the ligands that were previously listed as problematic. The downsampling results in 7970 protein-ligand pairs and 92 unique protein binding pockets in the cross-docking dataset, where in no case is a ligand paired to its cognate receptor. Notably, 2 pockets were removed (ALDR and CP3A4) due to all of the ligands either failing the filtering, or being marked as problematic. We evaluate the docking performance differences for various fractions of the total complexes per pocket for both Gnina and Smina to ensure the downsampling does not bias the performance of either software (Additional file 1: Fig. S1).

### Evaluation metrics

**TopN**: We evaluate docking performance by examining the output poses. The Open Babel *obrms* tool [42, 43] is used to determine the RMSD from each output pose to the binding pose. In redocking, the binding pose is defined by the crystal pose. In cross-docking, we define the binding pose as the conformation of the ligand when the cognate protein structure is aligned with the reference structure. If the RMSD to the known binding pose is less than 2 Å then we consider the pose to be “good.” The percentage of systems with a good pose ranked in the top N (TopN) is reported for redocking. In the case of cross-docking, we consider all poses of a ligand across an ensemble of non-cognate receptors. We calculate the TopN for each target individually and report the average across the 92 systems. Averaging is performed to avoid over-representing targets with a large number of ligands. This metric can be computed for any number of output poses, computing only the top pose is Top1.

**Avg Time Per System:** Properly benchmarking the time for the various CNN scoring options on both the redocking and cross-docking datasets would take a significant amount of time, so we utilize a filtered version of the PDBbind v.2016 [54] core set to determine runtime. This set consists of 263 complexes. The average runtime is calculated per system using the hyperfine benchmarking tool [55], computing the runtime for a minimum of 5 docking runs for each system (additional runs are automatically performed by hyperfine if high variability is encountered). We then average docking runtime over the whole PDBbind core set, to provide the average time for one docking run of the PDBbind core set. Timing evaluations were done on a dual 16-core 2.3 GHz Intel Xeon 5218 with 96 GB of RAM and a 11 GB RTX 2080 Ti GPU using CUDA 10.2 and cuDNN 7.6.5. All benchmarking runs were done on an otherwise unloaded system with four cores requested (cpu=4).

### Default model selection

When using CNN scoring, the user can utilize a single CNN model or an ensemble of CNN models where the final score is an average of each CNN model’s score. It has been shown that ensembles of predictors improve performance over a single predictive model [56]. However, due to the high computational cost of applying CNN models in comparison to empirical scoring functions, it is desirable to select a subset of the available models that provides improved scoring while limiting computational cost. The default CNN model ensemble was selected using a greedy forward algorithm. The ensemble was built in an iterative process using the “rescoring” option for CNN scoring so as to minimize the computational time for each iteration. In each round of the selection, models were chosen for their Top1 performance. We evaluate all of the versions of each CNN model (i.e. dense, dense_1, dense_2, dense_3, dense_4). In the first round of selection, all of the CNN models were tested for their individual ability to predict Top1 on both the cross-docking and redocking datasets. The next round of selection required testing of all two-model combinations with the model selected in the first step. Model selection continued, exploring all possible combinations of the built-in CNN models. The selection process concluded after five CNN models were selected for inclusion in the default ensemble.

### Default CNN scoring method

Gnina allows the usage of CNN scoring in various steps of the molecular docking process (Fig. 1). The CNN scoring option allows the user to change how the CNN is used to evaluate a ligand pose. If the CNN is not used at all in the scoring process (“none” option), then the molecular docking pipeline is essentially the same as Smina. The “rescoring” option has the lowest computational cost of the options that utilize the CNN models. With this option CNN models are used to score and re-sort the ligand conformations selected and refined by the non-CNN scoring function, defaulting to the Vina scoring function. For additional computational cost, the “refinement” option can be specified to use the CNN models for the refinement of the ligands after they have been selected by the Monte Carlo chains. In addition to refining the ligand conformations, the CNN models are used to score and resort the output poses, as the “rescore” option does. However, the Monte Carlo chains continue utilizing the non-CNN scoring function. The “all” option uses the CNN models as the scoring function throughout the course of the molecular docking procedure and has the highest computational cost by orders of magnitude. The CNN model is used for the selection process within the Monte Carlo chains, the refinement process after Monte Carlo selection, and the scoring and resorting of the poses before output. This option is very computationally intensive as the CNN is regularly queried for the energy of a particular conformation during the Monte Carlo sampling procedure.

In addition to the values allowed for cnn_scoring, the user is provided with cnn_empirical_weight to combine the non-CNN and the CNN scoring functions. Using mix_emp_force the refinement of the ligand poses can be computed with a linear combination of the CNN gradients and the non-CNN scoring functions force. mix_emp_energy uses the same linear combination of the scoring functions for computing the score of a given pose. The weighting of the Vina scoring function within the linear combination is selected with cnn_empirical_weight (default 1.0).

The default usage of the CNNs within the Gnina docking pipeline demands high accuracy while limiting computational costs. Therefore, each of the CNN scoring options is evaluated for both docking performance and runtime. Docking performance can be calculated on both the redocking and cross-docking datasets via TopN. To this end, Gnina is used with one of the CNN scoring options with the default CNN ensemble selected above to compute 9 ligand poses for each protein ligand system. We also investigate various values of cnn_empirical_weight while using the “refinement” option to determine if a linear combination of the non-CNN and CNN scoring functions provides greater docking performance. We use both mix_emp_energy and mix_emp_force with the cnn_empirical_weight parameter to use the linear combination of Vina and the default CNN ensemble for both refinement of poses and final scoring.

### Parameter exploration

Gnina has many parameters that alter the molecular docking pipeline (Fig. 2). A default value is found for each of these parameters to provide the best all around default behavior. In exploring the various values of the parameters we find the optimal value for both the redocking and the cross-docking datasets. Additionally, we also consider that the user may not know the exact location of the binding pocket on the receptor and may have to use the entire protein as the autobox for the docking pipeline. Therefore, our setting exploration considers the case in which the specific binding pocket is known and the case in which the whole protein is used to define a binding box. Some parameters directly impact the sampling procedure, such as exhaustiveness, autobox_add, num_modes, num_mc_saved, and min_rmsd_filter which are described below. These parameters control, to some extent, the extensiveness of the search during the Monte Carlo sampling procedure. As previously stated, exhaustiveness determines the number of Monte Carlo chains run during the sampling procedure. autobox_add increases the size of the binding box that the Monte Carlo chains sample. num_modes determines the number of ligand poses output by Gnina at the completion of the docking procedure. This is separate from num_mc_saved which defines the number of ligand poses saved for each Monte Carlo chain. The number of ligand poses retained after all of the Monte Carlo chains are completed is determined by either the number of modes or the number of Monte Carlo saved, whichever is larger. After all of the Monte Carlo chains have completed and the poses have been refined and sorted, the RMSD between all pairs of ligand poses is calculated. min_rmsd_filter removes one pose from a pair if the RMSD of the pair is less than the value of the parameter. This ensures the poses returned by the docking procedure are all different from one another. When using the CNN scoring function, another setting is how many different rotations of the protein-ligand complex the CNN is able to see for each conformation, cnn_rotation.

Evaluations for all of the parameters are carried out on both the redocking and cross-docking datasets. Each setting is varied individually using the default CNN ensemble determined above. The values explored for each parameter are defined in Tables 1 and 2. Values were explored around the previously set defaults for each of the parameters. Each value produced a set of poses, which was used to calculate a TopN.

**Table 1:** Parameters explored when the binding pocket has been defined

Argument	Description	Values explored
exhaustiveness	Number of Monte Carlo chains	4, 8, 16
autobox_add	Increase size of binding box	2, 4, 6, 8
num_modes	Number of output conformations	9, 100
num_mc_saved	Number of conformations saved from each Monte Carlo chain	20, 40, 60, 80, 100
min_rmsd_filter	Minimum RMSD to filter saved poses	0.5, 1.0, 1.5
cnn_rotation	Number of rotations of data to show the CNN	0, 1, 5, 10, 20

**Table 2:** Parameters explored when the binding pocket is not known and the whole protein is used for docking

Argument	Description	Values explored
exhaustiveness	Number of Monte Carlo chains	8, 16, 32, 64

### CNN scoring performance

All of the CNN models were trained on some subset of the cross-docking and redocking datasets. Generalization can be evaluated by determining the performance of the CNN scoring functions on the subset of the datasets that were not seen in training. This evaluation is carried out by removing any protein or ligand contained within the training data of the CNN models. All of the CNN models have been trained on different sets, so fully testing generality requires the removal of all of the proteins and ligands in the training set from both the redocking and cross-docking datasets. We removed all PDB IDs that were in the training sets of the CNN models [18, 45], leaving 441 and 178 protein-ligand pairs for the redocking and cross-docking sets, respectively.

The CNN models output both a CNNscore and a CNNaffinity for each of the conformations output by Gnina. CNNaffinity is the affinity of the docked complex as determined by the CNN, this metric has been evaluated in a previous work [45]. The CNNscore is a value between 0 and 1 that is used to rank the poses of the ligand, where a score of 1 denotes a perfect ligand pose. We would like to investigate if there is a correlation between high scores and low RMSD to the crystal pose. For ease of analysis, we only consider the top ranked pose. Using the top ranked pose for each complex, we investigate how filtering the poses by their CNNscore can affect the percentage of poses in which the RMSD to the crystal pose is less than 2 Å.

## Results

### Smina Comparison

Gnina is a fork of Smina that allows the utilization of CNN models as scoring functions. Therefore, without the use of the CNN models Gnina should function exactly as Smina does. However, unlike Smina, Gnina does computation with single (32 bit) precision rather than double (64 bit) precision due to the need to shift calculations to the GPU for efficient CNN scoring. Therefore, we ensure that the use of single precision does not negatively affect the docking power of the pipeline. The effect of this precision change can be evaluated by running Gnina without using any CNN scoring and with autobox_extend turned off, allowing us to compare to Smina docking results. We consider only redocking results as cross-docking results would require significantly more computation, and identification of differences due to precision can be done using only redocking results. Results for redocking do not show a significant difference for the output poses. A majority of the output poses are exactly the same, with slight differences seen for some output poses (Additional file 1: Figs. S2 and S3).

### Default model selection

The iterative process for construction of the default CNN ensemble, denoted Default Ensemble, is shown in Additional file 1: Table S1. The five selected models are dense, general_default2018_3, dense_3, crossdock_default2018, and redock_default2018. We now evaluate the docking performance boost that this ensemble provides over any single CNN model type (e.g. crossdock_default2018, dense, etc.) or an ensemble of the same CNN model type (e.g. crossdock_default2018_ensemble, dense_ensemble, etc.). We compare the CNN model(s) docking performance by evaluating TopN on both the redocking and the cross-docking tasks. The CNN models are used in the “rescoring” option for the CNN scoring to output 9 ligand conformations.

**Fig. 3:**
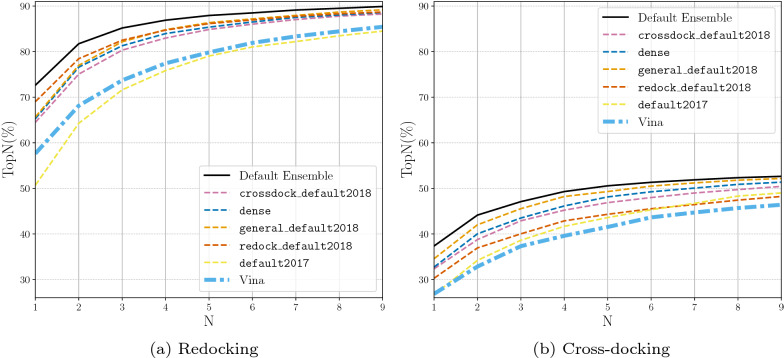
Docking using the single CNN models and the newly selected Default Ensemble for rescoring the output poses. The binding pocket is defined by the known binding ligand. TopN is the percentage of targets ranked above or at N with a RMSD less than 2 Å

The docking performance of the Default Ensemble is compared to the single model options in Fig. 3. While nearly all models are able to outperform Vina, we can see that the newly selected Default Ensemble significantly outperforms all of the single models on both the redocking task and the cross-docking task.

**Fig. 4:**
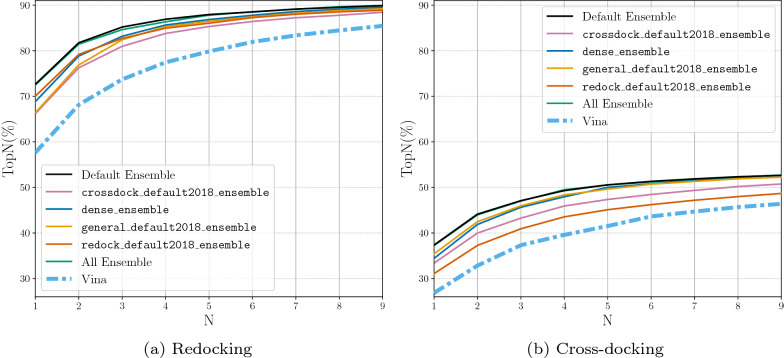
Docking using the ensemble of each type of CNN model, the full ensemble of CNN models, and the newly selected Default Ensemble for rescoring the output poses. The binding pocket is defined by the known binding ligand. TopN is the percentage of targets ranked above or at N with a RMSD less than 2 Å

**Fig. 5:**
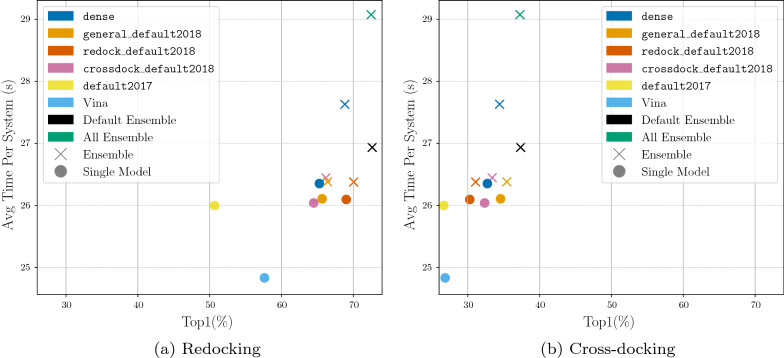
Evaluation of the average time to dock one protein-ligand system from the PDBbind core set v.2016. Top1 is the percentage of top ranked targets with a RMSD less than 2 Å

When the Default Ensemble is compared to the ensembles of each of the individual CNN model types, we see that the Default Ensemble is able to outperform all of the ensembles composed of one model type (Fig. 4). The ensemble selection procedure determined five CNN models whose combined performance on ranking low RMSD poses first beats the performance of the ensemble utilizing all of the built-in models while significantly reducing computational cost. The All Ensemble is the ensemble composed of all 16 CNN models built-in to the Gnina software. The Default Ensemble is able to meet the docking performance of this large ensemble, even when considering cross-docking results per pocket (Additional file 1: Fig. S4), while only being composed of five models. Reducing the number of models in the ensemble enables the computations to be several seconds faster for an average docking run (Fig. 5). This reduction is likely due to the inclusion of only two of the dense models which take the longest to run because of the high number of parameters in the models. The computational speedup afforded by the Default Ensemble over the All Ensemble increases when no GPU is used for docking (Additional file 1: Table S2). The computational speed boost can have a significant impact when performing a large number of docking runs or when there is no GPU available for enhanced parallelism of the scoring computation.

When comparing Figs. 3 and 4 we can see that the ensembles composed of the individual model types are able to outperform their single model counterparts. We therefore omit the single models for the remaining evaluations.

### Default CNN scoring method

**Fig. 6:**
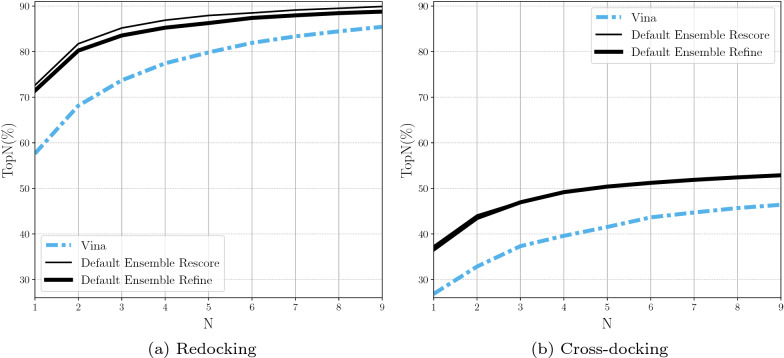
Comparing the Default CNN Ensemble for use in only rescoring of the poses output by the Monte Carlo chains or the refinement of the poses followed by a rescoring of the poses. The “refine” option has nearly the same docking performance as the “rescore” option when cross-docking. TopN is the percentage of targets ranked above or at N with a RMSD less than 2 Å

We evaluate the performance of the Default Ensemble with the “rescore,” and “refinement” options of cnn_scoring. The usage of the “all” option was unable to complete on the PDBbind Core set in a reasonable amount of time, so it was not considered. The “all” option requires usage of the CNN scoring function for every mutation of the ligand within every Monte Carlo chain with each usage of the CNN scoring function having a high computational cost. This leaves us with the “refinement” and “rescoring” options of cnn_scoring. Figure 6 shows the Default Ensemble performs nearly as well with either option. We can also see that using cnn_emp_weight with both mix_emp_energy and mix_emp_force does not significantly alter the docking performance when using the “refinement” option (Additional file 1: Figure S5).

However, from looking at the average time to perform molecular docking for one system we see that “refinement” takes an order of magnitude longer than “rescoring” (Additional file 1: Figure S6). Time for performing “rescoring” on an average system is similar to the time to perform docking with the Vina scoring function. We find it reasonable to use “rescore” as the default option for the CNN scoring due to its docking performance and runtime.

### Parameter exploration

**Fig. 7:**
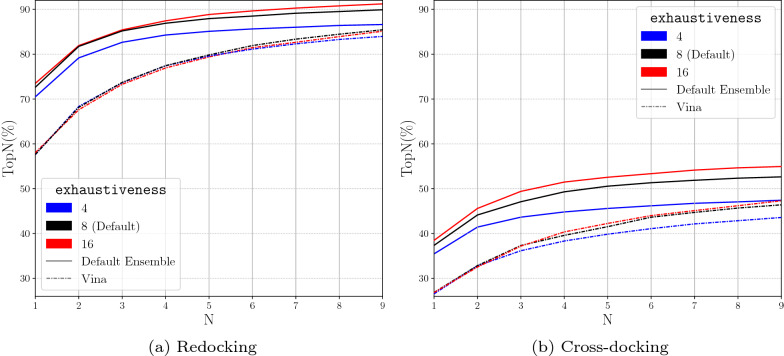
Evaluating the role of exhaustiveness in the performance of docking with the Default CNN Ensemble by analyzing TopN. TopN is the percentage of targets ranked above or at N with a RMSD less than 2 Å

Changes in the exhaustiveness alter the amount of sampling that occurs. When the exhaustiveness is increased, we see an increase in the performance of docking (Fig. 7). This is as expected as more Monte Carlo chains randomly mutating the ligand conformation provides the docking procedure with more opportunities to randomly sample the correct pose. However, there are no significant performance gains after a value of 8. An exhaustiveness of 16 provides some performance boost, but this boost may be accompanied by a doubling of the computational time. The Monte Carlo chains are evaluated in parallel, but parallelism is limited by the number of cores available to Gnina. If the exhaustiveness is greater than the CPUs provided to Gnina, the number of simultaneously running Monte Carlo chains is equal to the number of CPUs. Therefore, in typical usage of Gnina, an exhaustiveness level of 8 is sufficient for adequate performance levels while minimizing the computational load when targeting a specific binding site.

**Fig. 8:**
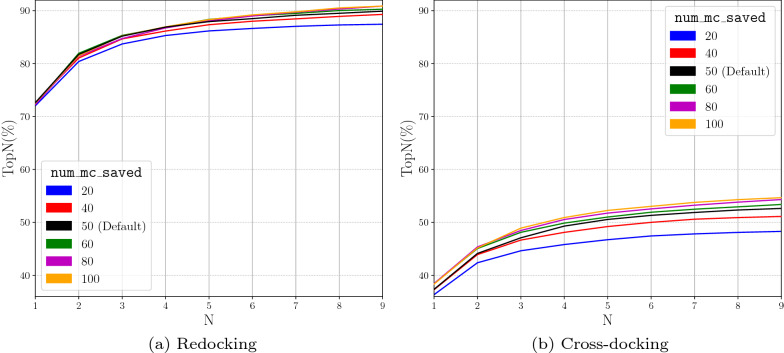
Evaluation of the Number of Monte Carlo Saved in the performance of docking with the Default CNN Ensemble by analyzing TopN. TopN is the percentage of targets ranked above or at N with a RMSD less than 2 Å

**Fig. 9:**
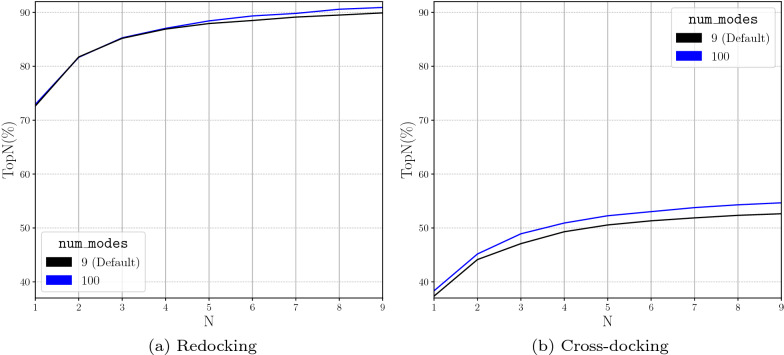
Evaluating a much greater number of modes on the performance of docking with the Default CNN Ensemble by analyzing TopN. TopN is the percentage of targets ranked above or at N with a RMSD less than 2 Å

Increasing the num_mc_saved parameter increases the chances of sampling accurate docking poses, as increasing the number of output conformations from each Monte Carlo chain increases the likelihood of finding the correct pose. However, this will also increase computational time due to the fact that more poses require refinement and final scoring. As num_mc_saved gets closer to 100, we see that the docking performance boost is reduced (Fig. 8). Therefore, we select a new default value of 50 for num_mc_saved to minimize the computational overhead while still increasing the performance of the docking routine. The num_mc_saved and num_modes affect one another; the number of poses saved from each Monte Carlo chain is the maximum of the two values. When looking at the first 9 poses, we see an increase in the docking performance with a substantially greater value for the number of output poses (num_modes) (Fig. 9). This is due to num_modes forcing each Monte Carlo chain to output a number of poses greater than num_mc_saved. Increasing the default value of num_modes to a value higher than 50 (the default value for num_mc_saved) will again increase computational overhead, so the default value is set to 9.

We next evaluate the size of the targeted binding site (box). The autobox_add parameter increases the search space for the docking program to be larger than the rectangular prism defined by the autobox_ligand input to Gnina. In redocking (Additional file 1: Figure S7a), the expansion of the search space decreases performance as the expanded search space increases the potential conformational space of the ligand. A higher value of autobox_add is necessary to ensure that the correct binding site, which may differ from the binding site of the autobox_ligand, is included in the search space. This is shown during cross-docking (Additional file 1: Figure S7b), where only the binding site of the cognate ligand is known. A low value of autobox_add artificially improves docking performance when the binding pose is known by unrealistically constraining the search space. We therefore select a default value of 4 for autobox_add to keep the box small while still providing room for error in the selection of the binding site.

Changes to the CNN rotations do not significantly change the scoring of the Default Ensemble (Additional file 1: Figure S8). The CNN Ensemble is able to determine the correct score for the ligand pose regardless of the rotation of the ligand and protein complex. Altering the value of the minimum RMSD filter does not change the results of the docking (Additional file 1: Figure S9). Filtering out poses with similar conformations increases the diversity of poses that the CNN ensemble ranks. However, the CNN ensemble is able to accurately rank the poses it sees, providing high scores to poses with low RMSD to the known binding pose.

### Whole protein docking

**Fig. 10:**
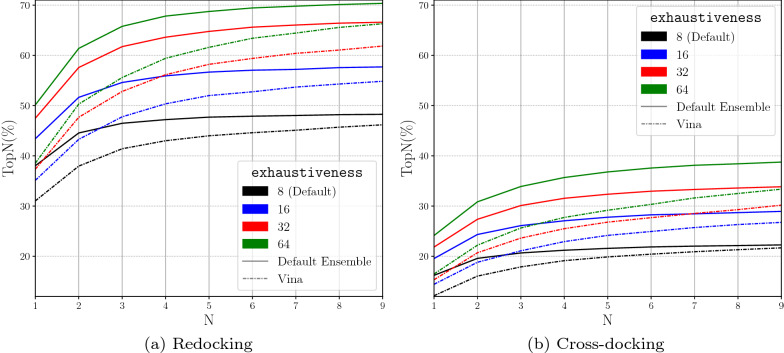
Increasing the exhaustiveness when using the whole protein as the binding box. TopN is the percentage of targets ranked above or at N with a RMSD less than 2 Å

Next, we evaluate the performance of docking when using the whole protein as the defined binding site. Whole protein docking can be used for new protein targets when the true binding site is unknown. The performance of docking is expected to be reduced as the sampling space has been significantly increased. When using the whole protein for the sampling space, the ligand tends to get stuck at local energy minima which are distant from the actual binding site. Most of the protein surface is not hospitable for ligand binding, so once a potential pocket is discovered, exploring more of the protein surface has a low probability during the Monte Carlo sampling procedure. Comparing Figs. 10 to 7 shows that the docking performances of both Vina and the Default CNN Ensemble are reduced from when the binding pocket was explicitly defined. Redocking performance decreases from 73% to 38% at Top1 with exhaustiveness at 8 while cross-docking decreases from 37% to 16% at Top1 with exhaustiveness at 8 (cross-docking per pocket Top1 is shown in Additional file 1: Figure S10). The larger potential docking space requires more sampling to find a ligand conformation with low RMSD to the known binding pose. As expected, with whole protein docking we see greater increases in performance with increased sampling (exhaustiveness). By again comparing Figs. 10 with 7 we see that this boost in performance is larger than when the binding pocket is defined explicitly and does not exhibit the same diminishing returns. When increasing exhaustiveness from 8 to 16 in whole protein docking Top9 increases from 48% to 58% and 22% to 29% for redocking and cross-docking, respectively. If the binding pocket is explicitly defined, the same change in exhaustiveness only increases Top9 from 87% to 88% and 53% to 55% for redocking and cross-docking, respectively. For this reason, when performing whole protein docking, we recommend setting the value of exhaustiveness as high as possible given time constraints.

### Flexible docking

We now test the performance of Gnina for docking with flexible side chains. Since receptor flexibility is only useful in the context of cross-docking, we limit our tests to the cross-docking dataset; allowing side-chain re-arrangements would only deteriorate the performance in redocking given that the receptor is already in the correct conformation.

Flexible docking is computationally more expensive than docking with a rigid receptor because of the larger number of degrees of freedom to be sampled. For this reason, we only use the default parameters carefully selected above. In order to define the side chains to be treated as flexible, autobox_ligand is also used as flexdist_ligand and flexdist is set to 3.5 Å, which gives a reasonable representation of the protein-ligand binding site [57]. Therefore, conformations for all side chains with at least one atom within 3.5 Å from flexdist_ligand are sampled during docking.

Out of the 7970 systems in the cross-docking dataset, flexible side chains are not identified for 24 protein-ligand complexes (see Additional file 1: Table T3 for details). Such systems are discarded from the following analysis of flexible docking since they are equivalent to rigid docking. Additionally, 9 systems were problematic when computing RMSDs for the flexible residues because of connectivity issues. Disulfide bonds between cysteine residues are allowed to break during sampling (with a software warning), which results in a different connectivity in the output file for four systems. In the other five systems, the connectivity between flexible residues in the input and output file was found to be different and therefore they were discarded (see Additional file 1: Table T3 for details). All nine systems with connectivity issues were removed from the analysis, resulting in a dataset of 7937 cross-docked complexes.

In order to assess the performance of flexible docking compared to rigid docking, we look at RMSD differences between ligands docked with both methods in relation to the similarity between binding pockets of the cognate and docking target receptors. The similarity of the binding pockets is assessed via side chains RMSD between the docking target (non-cognate receptor) and the cognate receptor, which we denote target-cognate RMSD. This is distinct form either the side chain RMSD between docking input and output (target-pose RMSD) and the side chain RMSD between cognate receptor and docking output (cognate-pose RMSD). The target-cognate RMSD is computed by finding the best match between residues in the target and cognate receptors that are within 3.5 Å from the ligand being docked.

**Fig. 11:**
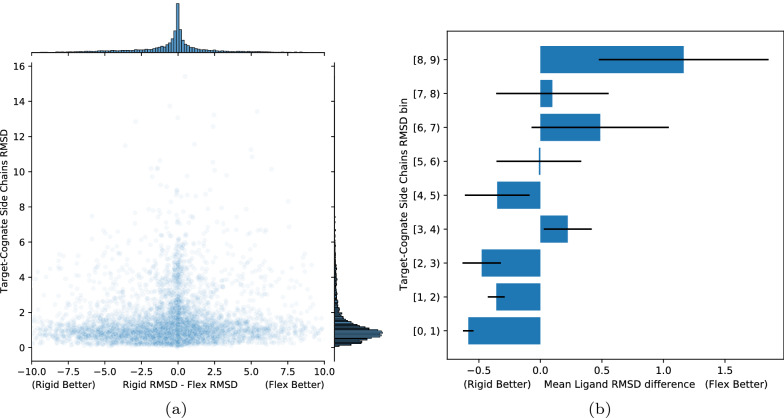
Comparison between rigid and flexible docking with the default Gnina parameters: (**a**) ligand RMSD differences between rigid and flexible docking versus target-cognate side chain RMSDs, (**b**) average ligand RMSD difference for different 1 Å intervals of target-cognate side chains RMSD

Figure 11a shows the difference in RMSDs for the ligand top pose between flexible and rigid docking versus the target-cognate RMSD. For higher target-cognate RMSDs, indicating differences in the binding pocket between the target and cognate receptor, one would expect flexible docking to perform better. However, as we can see, the difference in ligand RMSD between flexible and rigid docking for the top pose varies widely between systems and there in no clear advantage in flexible docking. The overall RMSD distributions for the ligand top poses (Additional file 1: Figure S11) are fairly similar, with slightly more systems with low RMSD for rigid docking than flexible docking.

Figure 11b shows the average RMSD difference between rigid and flexible docking for different 1 Å intervals of target-cognate side chains RMSDs. For low target-cognate RMSDs, which correspond to a highly similar binding site and therefore a situation akin to redocking, rigid docking seems to be advantageous on average, as expected. In the other hand, for higher target-cognate side chains RMSDs, indicating a lower similarity between binding sites, the situation is less clear. Flexible docking seems to be equivalent or slightly more advantageous, on average, especially at higher target-cognate side chains RMSDs. However, as it can be inferred from Fig. 11a, the number of systems with target-cognate side chains RMSD higher than 6 Å is low and therefore the apparent improvement in ligand RMSD for flexible docking is inconclusive.

When performing flexible docking, the side chains identified as flexible are included in the calculation of the bounds for the box defining the search space. This results in a larger search space for flexible docking compared to rigid docking, which in turn might require a higher exhaustiveness for better sampling (although by default the number of Monte Carlo steps is already proportional to the number of degrees of freedom, hence the increased computational cost of flexible docking). It is also worth stressing that we used the default CNN models, which were not explicitly trained for flexible docking.

Given the much higher computational cost of flexible docking, the optimization of Gnina default parameters and training of new CNN models for this specific task is outside the scope of the present work and will be addressed in future versions of Gnina. However, it is clear that improvements for flexible docking are system-dependent and, therefore, accounting for the increased computational cost, it is reasonable to use rigid docking as the default docking method.

### CNN scoring performance

**Fig. 12:**
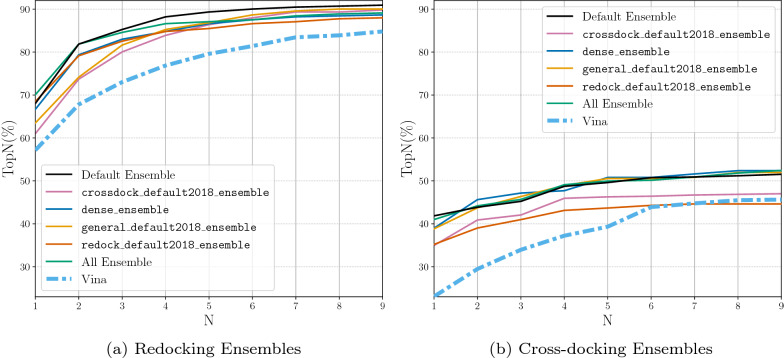
CNN model ensembles evaluated on the subset of proteins and ligands not present in their training datasets. Ensemble models used with the default arguments defined above. TopN is the percentage of targets ranked above or at N with a RMSD less than 2 Å

We evaluate all of the CNN models on a subset of the data that was not seen during training to ensure that the CNN models are able to generalize to unseen protein-ligand systems. We also show the Vina results for the same subset of protein-ligand systems. The CNN models’ docking performance decrease relative to the full sets when looking at the top pose, (comparing Figs. 12 to 4). Redocking Top1 performance drops from 73% to 68% on the Default Ensemble for the filtered set and the full set, respectively, while Vina remains at about 57%. Cross-docking Top1 performance increases from 37% to 42% for the Default Ensemble and decreases from 27% to 23% when using the Vina scoring function. Overall, the Default Ensemble still shows a strong docking performance boost when used to rescore poses rather than using the Vina scoring function to score poses even on protein-ligand systems not seen during training.

Finally, we examine the importance of the output CNNscore as a measure of the confidence of the prediction.

**Fig. 13:**
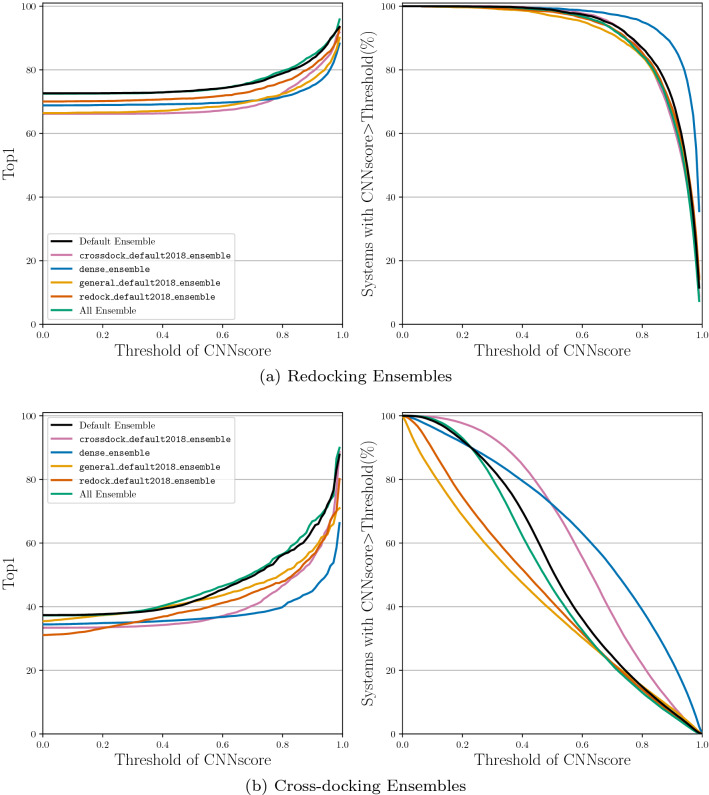
Thresholding the top pose by the score determined by the CNN. Top1 is the percentage of top ranked targets with a RMSD less than 2 Å

We show in Fig. 13 that poses with high CNNscores are more likely to be low RMSD to the known binding pose. However, when the CNNscore threshold is close to 1, each CNN ensemble has few poses remaining. Comparing the percentage of complexes remaining when thresholding by CNNscore, we can see that the CNN models are much more confident in the poses when they are performing the redocking task: 87% of systems have a top pose with a score higher than 0.8. All of the CNN ensembles identify fewer poses with a high CNNscore for the cross-docking task: only  15% of cross-docked ligands have a pose with a score higher than 0.8 (Additional file 1: Figure S12). In general, higher CNNscores imply that a pose is more likely to have a low RMSD.

## Discussion

We show that our computational docking software Gnina is able to outperform AutoDock Vina scoring by using CNN models to rescore generated poses. Without using a CNN model, our software is equivalent to using the Smina docking software, which is a fork of Vina. Gnina allows the user to utilize CNN models as scoring functions within the docking pipeline in a variety of ways. The various CNN scoring options allow the specified CNN models to replace the scoring function in the sampling, refinement, and rescoring steps of the docking pipeline. We first establish an ensemble of built-in CNN models to be used as the default ensemble. This ensemble of models is selected for its docking and runtime performance. The selected ensemble, termed the Default Ensemble, is able to exceed the ranking performance of Vina (Top1 increases from 58% to 73% and from 27% to 37% for redocking and cross-docking, respectively) while only adding two seconds to the average compute time when utilizing GPUs. It also significantly outperforms any single model or any ensemble of identical models while being significantly faster than an ensemble of all models. The Default Ensemble performs almost equally well when performing refinement of the sampled poses rather than using the Vina scoring function. Refinement with the CNN models is not recommended as the compute cost is significant and the performance is less than simply using the CNN models to rescore the poses refined by the Vina scoring function.

We next derive the default parameters when using the Default Ensemble for docking with Gnina. autobox_add increases the amount of space around the defined binding pocket to allow more volume for sampling to investigate. Increases in autobox_add decrease the accuracy of the predicted binding pose when the precise binding pocket is known. However, a higher autobox_add is necessary if the true binding site is not known. We find that increases in exhaustiveness, or the number of Monte Carlo chains run, boost the performance of the docking procedure at the cost of extra computation. We also show that our Default Ensemble with an exhaustiveness value of 8 (Top1 73% on redocking and 37% on cross-docking) is able to outperform the Vina scoring function with double the exhaustiveness (Top1 58% on redocking and 27% on cross-docking). This shows that using the Default Ensemble for rescoring is more valuable than doubling the amount of sampling of the docking procedure. Similarly, increasing the number of poses saved from each Monte Carlo chain, num_mc_saved, and the number of poses output by the docking pipeline, num_modes, increases the performance of the docking routine at the expense of increased computation. The value of the cnn_rotation and the value of min_rmsd_filter do not seem to alter the performance of the docking pipeline. The default arguments for Gnina when the binding pocket is explicitly defined are: autobox_add (4), exhaustiveness (8), num_mc_saved (50), num_modes (9), min_rmsd_filter (1.0), and cnn_rotation (0).

However, when the exact binding pocket is not known and the whole protein is used as the defined binding pocket we see that exhaustiveness has a much greater impact on performance. We see that the Default Ensemble with exhaustiveness 16 (Top1 43% on redocking and 20% on cross-docking) is able to outperform the Vina scoring function even when the exhaustiveness is quadrupled to 64 (Top1 38% on redocking and 16% on cross-docking). Again, this shows the importance of using the Default Ensemble for rescoring rather than increasing exhaustiveness with the Vina scoring function. The Default Ensemble still shows docking performance improvements with increasing exhaustiveness on both redocking and cross-docking when the exact binding pocket is unknown. We therefore recommend the largest value of exhaustiveness that can be used given time constraints when performing whole protein docking.

We tested the impact of performing cross-docking with flexible side chains (flexible docking). For high binding pocket similarity, flexible docking resulted in a slight deterioration of average ligand top pose RMSDs, as expected. However, for low binding pocket similarity there is no clear advantage for flexible docking, whose success is very much system-dependent. In light of this and given the much higher computational cost, rigid docking remains the most suitable default docking procedure.

Finally, we evaluate the ability of the CNN ensembles to score ligand conformations. In order to ensure that our CNN models are generalizing to unseen protein-ligand complexes, we filter the redocking and cross-docking benchmark datasets to only include protein-ligand pairs that were not seen during training of the CNNs. We show that our CNN model ensembles are able to outperform the Vina scoring function. The CNN models are able to generalize to unseen examples and properly score the ligand conformations such that the low RMSD poses are ranked higher more often than when using the Vina scoring function. The score output can provide a probability of a pose being less than 2 Å from the binding pose. When the CNN outputs a score greater than 0.8 during cross-docking there is at least a 56% probability that the pose is less than 2 Å RMSD from the correct pose. If redocking is performed, then this probability is 79%. The score output by the CNN models can be used as an indicator of the confidence in the quality of the generated ligand conformation; however, particularly when cross-docking, high scoring poses are less common (Fig. 13b).

Gnina demonstrates significant improvements over a popular empirical scoring function, but many challenges remain. The failure of using gradients from the CNN scoring to meaningfully improve docking results points to the need to train new models specifically for this task. Such models, by learning a smoother energy surface than a classical scoring function, may significantly reduce the need for extensive sampling and make full CNN docking more computationally tractable. Additionally, new models trained specifically for the flexible docking task would likely improve performance in this area. Gnina 1.0 provides a solid framework for these and other improvements. We look forward to receiving contributions and feedback from the community to further enhance the software in future releases as part of our open development process.

## Supplementary Information

**Additional file 1:** **Figure S1**: Analyzing the effect on docking performance when downsampling the cross-docking dataset to include a fraction of the protein-ligand pairs per pocket. **Figure S2**: Comparison of the RMSD(Å) of the top 3 poses output by Gnina with no CNN in the docking pipeline and Smina. **Figure S3**: Comparison of the minimum and maximum RMSD(Å) poses output by Gnina with no CNN in the docking pipeline and Smina. **Figure S4**: Cross-docking results using a defined binding pocket. **Figure S5**: Evaluating different values of cnn emp weight on docking performance when using the Default Ensemble. **Figure S6**: Time to perform one docking run when using the Default Ensemble for “rescore” or refine” in comparison to only using the Vina scoring function. **Figure S7**: Evaluating the effect on docking performance when the value of autobox add is changed while using the Default Ensemble for rescoring. **Figure S8**: Evaluating the effect on docking performance when the value of cnn rotation is changed while using the Default Ensemble for rescoring. **Figure S9**: Evaluating the effect on docking performance when the value of min rmsd filter is changed while using the Default Ensemble for rescoring on both the redocking and cross-docking datasets. **Figure S10**: Cross-docking results using the whole protein as the defined binding pocket. **Figure S1**: Ligand RMSD distributions for the top pose in the cross-docking dataset, for both flexible and rigid docking. **Figure S12**: Thresholding the cross-docking results by CNNscore and evaluating Top1(%) per pocket. A pose is retained if the CNN score is greater than the value on the x-axis. Grey cells indicate that no poses are left in the pocket. **Table S1**: Optimal Model Ensemble Selection. Performance given by Top1, the percent of systems with RMSD less than 2 Å from the top pose to the known binding pose. **Table S2**: Average time to dock one protein-ligand system from the filtered PDBbind core set v.2016. Comparing runtime when GPU is used to when no GPU is used for docking. **Table S3**: Pocket, ligand and receptor identifiers for the systems excluded from the analysis of flexible docking, together with the reason for exclusion.

## Data Availability

*Project name:* Gnina. *Project home page:* https://github.com/gnina/gnina. *Operating systems:* Linux (Docker container available). *Programming language*: C++, CUDA. *Other requirements:* CUDA, Open Babel 3. *License:* GPL2/Apache License. *Any restrictions to use by non-academics:* None. All data and scripts used in the creation of this manuscript are available at https://github.com/dkoes/GNINA-1.0. Source code, scripts, and models are available via the project homepage, https://github.com/gnina.All data and scripts used in the creation of this manuscript are available at https://github.com/dkoes/GNINA-1.0. Source code, scripts, and models are available via the project homepage, https://github.com/gnina.
