# Supplementary material for: GNINA 1.0: molecular docking with deep learning
Source: J Cheminform. 2021 Jun 9;13:43. doi: 10.1186/s13321-021-00522-2 (PMC8191141; doi:10.1186/s13321-021-00522-2)
Supplement: Supplementary file 1 — Additional file 1: Figure S1: Analyzing the effect on docking performance when downsampling the cross-docking dataset to include a fraction of the protein-ligand pairs per pocket. Figure S2: Comparison of the RMSD(Å) of the top 3 poses output by Gnina with no CNN in the docking pipeline and Smina. Figure S3: Comparison of the minimum and maximum RMSD(Å) poses output by Gnina with no CNN in the docking pipeline and Smina. Figure S4: Cross-docking results using a defined binding pocket. Figure S5: Evaluating different values of cnn emp weight on docking performance when using the Default Ensemble. Figure S6: Time to perform one docking run when using the Default Ensemble for “rescore” or refine” in comparison to only using the Vina scoring function. Figure S7: Evaluating the effect on docking performance when the value of autobox add is changed while using the Default Ensemble for rescoring. Figure S8: Evaluating the effect on docking performance when the value of cnn rotation is changed while using the Default Ensemble for rescoring. Figure S9: Evaluating the effect on docking performance when the value of min rmsd filter is changed while using the Default Ensemble for rescoring on both the redocking and cross-docking datasets. Figure S10: Cross-docking results using the whole protein as the defined binding pocket. Figure S1: Ligand RMSD distributions for the top pose in the cross-docking dataset, for both flexible and rigid docking. Figure S12: Thresholding the cross-docking results by CNNscore and evaluating Top1(%) per pocket. A pose is retained if the CNN score is greater than the value on the x-axis. Grey cells indicate that no poses are left in the pocket. Table S1: Optimal Model Ensemble Selection. Performance given by Top1, the percent of systems with RMSD less than 2 Å from the top pose to the known binding pose. Table S2: Average time to dock one protein-ligand system from the filtered PDBbind core set v.2016. Comparing runtime when GPU is used to when no G [file 13321_2021_522_MOESM1_ESM.pdf]

Supporting Information: GNINA 1.0: Molecular docking with deep learning

Andrew McNutt,† Paul Francoeur,† Rishal Aggarwal,‡ Tomohide Masuda,† Rocco Meli,¶ Matthew Ragoza,† Jocelyn Sunseri,† and David Ryan Koes*,†

†*Department of Computational and Systems Biology, University of Pittsburgh, Pittsburgh, PA*

‡*Center for Computational Natural Sciences and Bioinformatics, International Institute of Information Technology, Hyderabad 500 032, India*

¶*Department of Biochemistry, University of Oxford, Oxford, United Kingdom*

E-mail: dkoes@pitt.edu

Table S1: Optimal Model Ensemble Selection. Performance given by Top1, the percent of systems with RMSD less than 2Å from the top pose to the known binding pose.

Iteration #	Model Selected	Redocking Top1(%)	Redocking Ranking	Crossdocking Top1(%)	Crossdocking Ranking
0	dense	67.7	5	37.8	1
1	general_default_3	70.4	6	39.8	2
2	dense_3	71.2	7	40.4	2
3	crossdock_default2018 0	71.7	7	40.6	2
4	redock_default2018	72.2	1	40.1	13

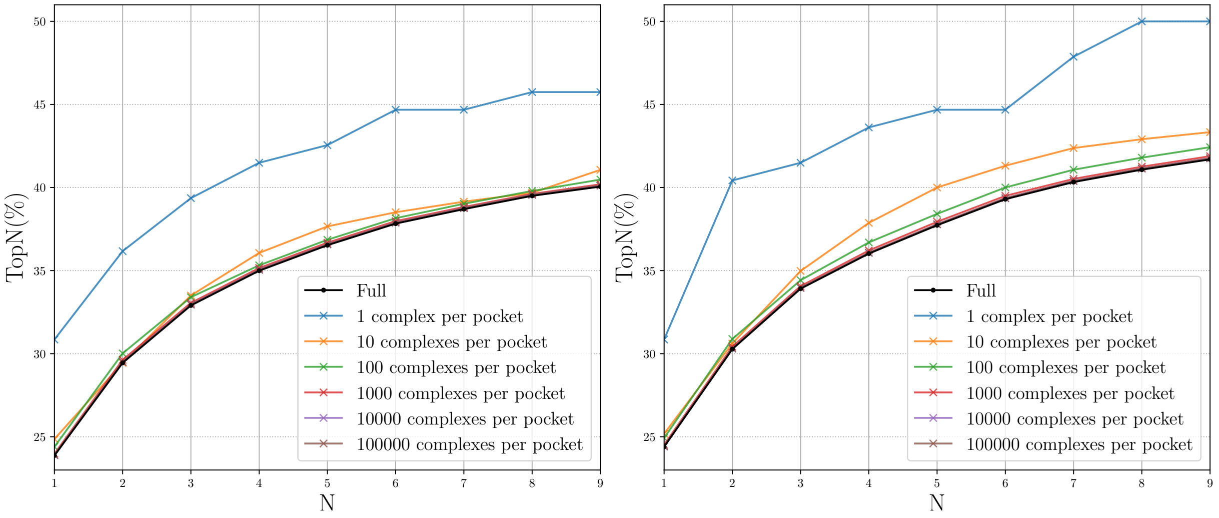

(a) SMINA (b) GNINA

Figure S1: Analyzing the effect on docking performance when downsampling the cross-docking dataset to include a fraction of the protein-ligand pairs per pocket. TopN is the percentage of targets ranked above or at N with a RMSD less than 2Å.

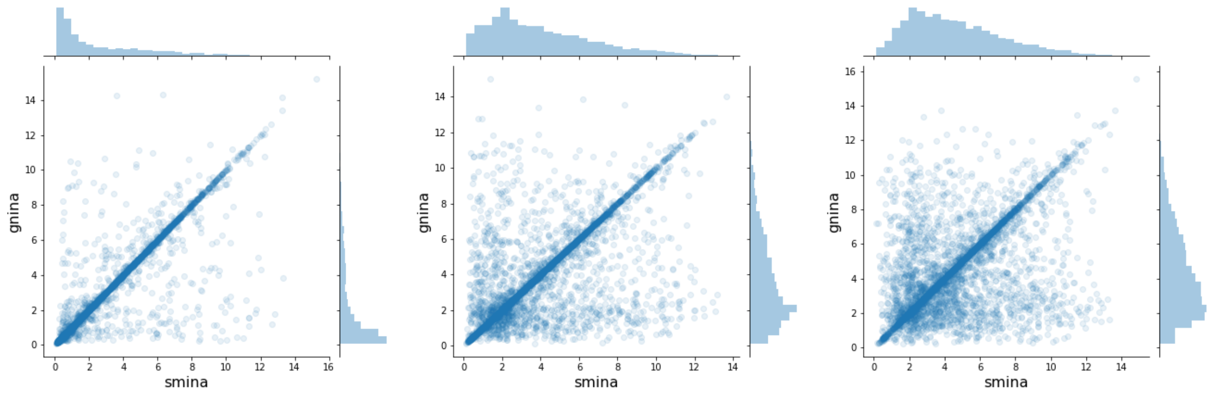

(a) First Pose (b) Second Pose (c) Third Pose

Figure S2: Comparison of the RMSD(Å) of the top 3 poses output by GNINA with no CNN in the docking pipeline and SMINA. Both docking software were run with the same arguments. Gnina was run with `autobox_extend` turned off.

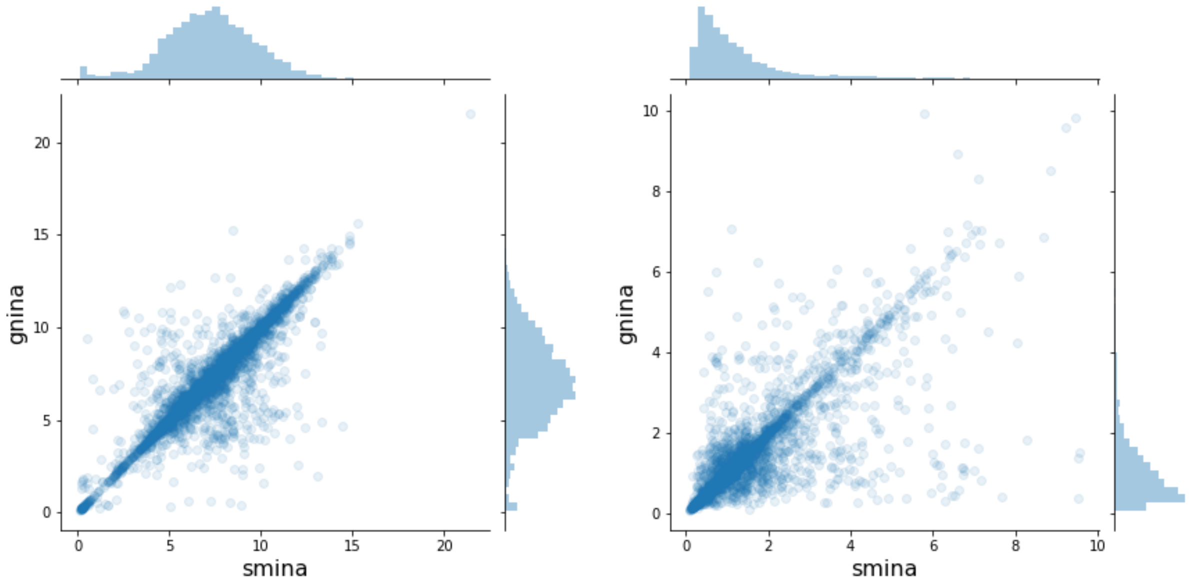

(a) Minimum RMSD(Å) Pose

(b) Maximum RMSD(Å) Pose

Figure S3: Comparison of the minimum and maximum RMSD(Å) poses output by Gnina with no CNN in the docking pipeline and Smina. Both docking software were run with the same arguments. Gnina was run with `autobox_extend` turned off.

Table S2: Average time to dock one protein-ligand system from the filtered PDBbind core set v.2016. Comparing runtime when GPU is used to when no GPU is used for docking. "rescore" option selected when a CNN is used.

Model Name	CPU only Average Docking Time (s)	GPU accelerated Average Docking Time (s)
Default Ensemble	194.38	26.94
`crossdock_default2018`	31.70	26.04
`crossdock_default2018_ensemble`	59.37	26.45
`dense`	76.71	26.35
`dense_ensemble`	449.45	27.63
`general_default2018`	31.71	26.11
`general_default2018_ensemble`	58.71	26.38
`redock_default2018`	31.82	26.10
`redock_default2018_ensemble`	59.00	26.38
`default2017`	32.70	26.00
All Ensemble	561.36	29.08
Vina	24.87	24.83

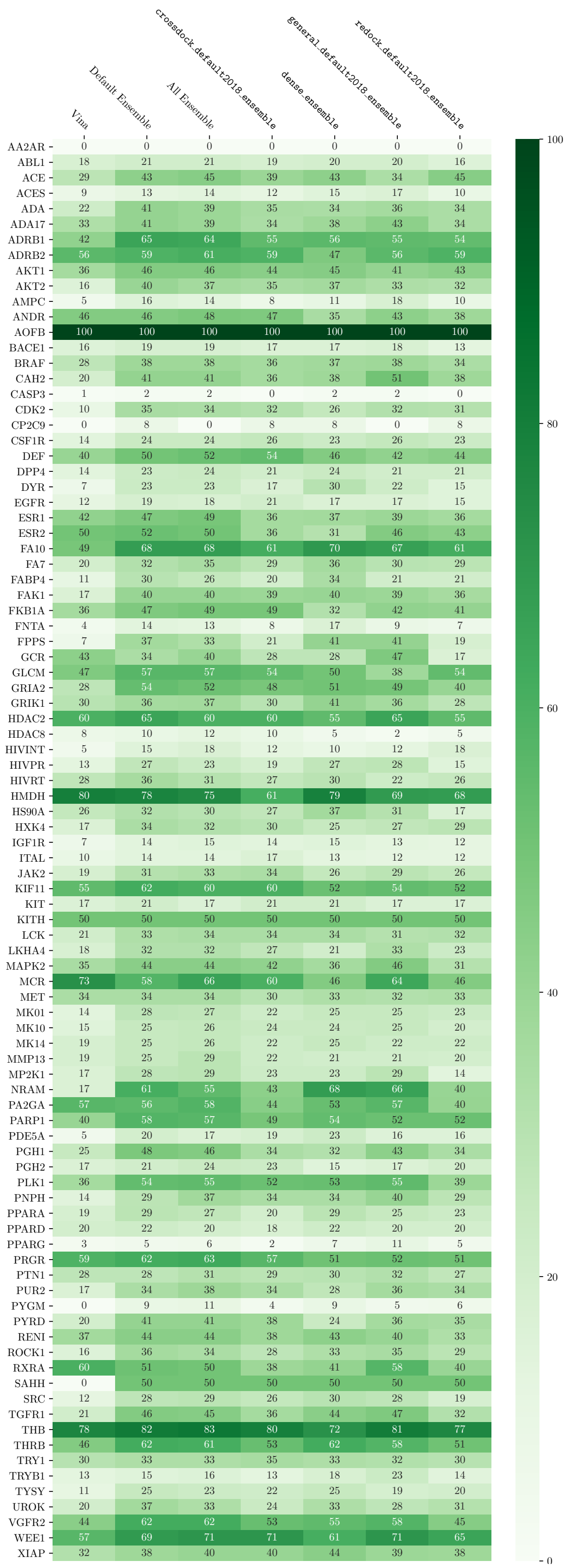

Figure S4: Cross-docking results using a defined binding pocket. Evaluating the Top1(%) per pocket. Top1 is the percentage of top ranked targets with a RMSD less than 2Å.

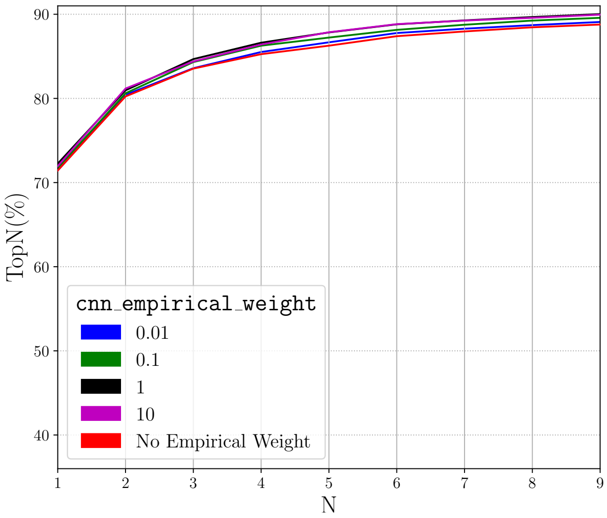

(a) Redocking

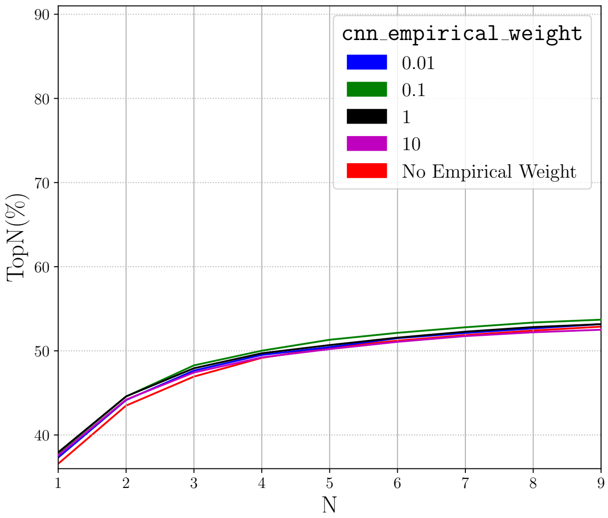

(b) Crossdocking

Figure S5: Evaluating different values of `cnn_emp_weight` on docking performance when using the Default Ensemble. Using "refinement" option for `cnn_scoring`. Both `mix_emp_energy` and `mix_emp_force` are used.

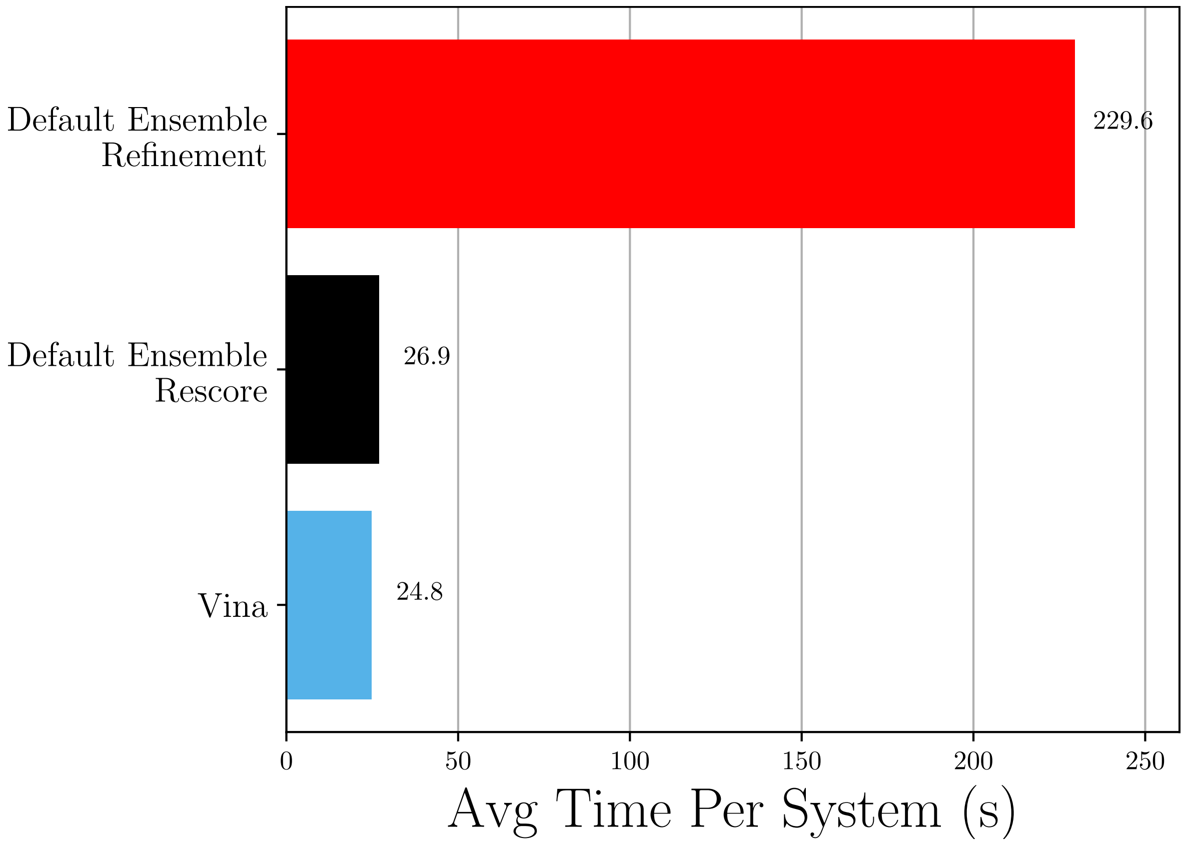

Figure S6: Time to perform one docking run when using the Default Ensemble for "rescore" or "refine" in comparison to only using the Vina scoring function.

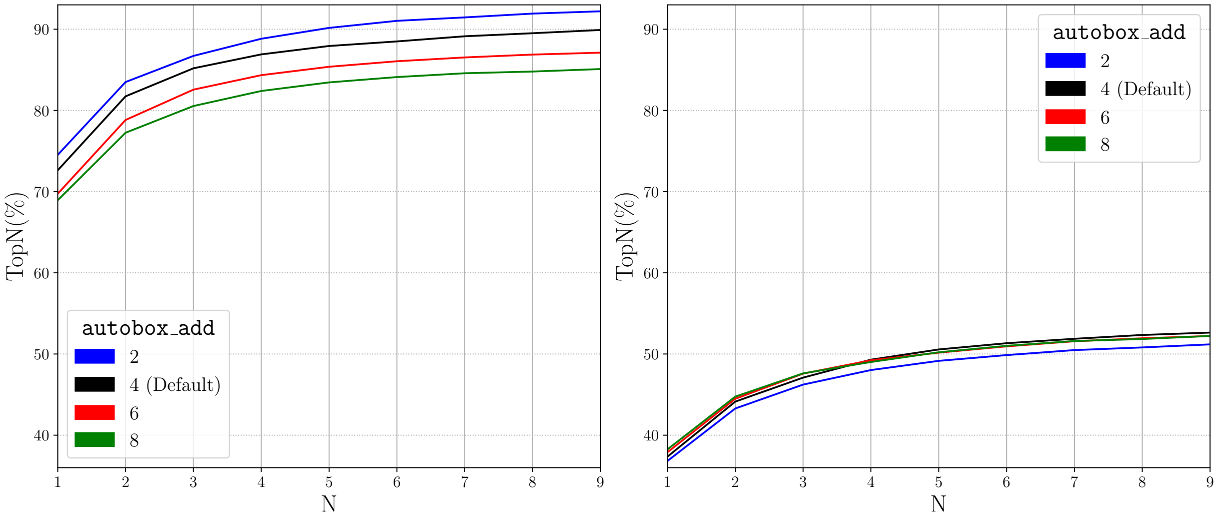

(a) Redocking

(b) Crossdocking

Figure S7: Evaluating the effect on docking performance when the value of `autobox_add` is changed while using the Default Ensemble for rescoring. TopN is the percentage of targets ranked above or at N with a RMSD less than 2Å

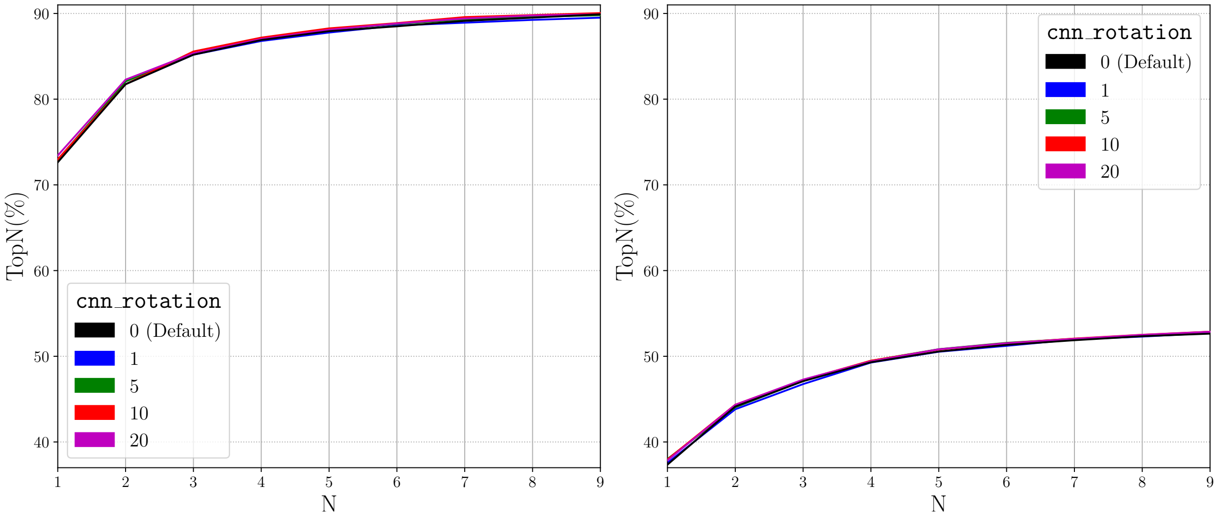

(a) Redocking

(b) Crossdocking

Figure S8: Evaluating the effect on docking performance when the value of `cnn_rotation` is changed while using the Default Ensemble for rescoring. When CNN rotations is set to 0 the CNN sees a randomly rotated grid of the ligand conformation. TopN is the percentage of targets ranked above or at N with a RMSD less than 2Å

Table S3: Pocket, ligand and receptor identifiers for the systems excluded from the analysis of flexible docking, together with the reason for exclusion. 24 systems are discarded since no flexible residues were identified. 5 systems were discarded because bonding information was different between input and output files, 4 systems were discarded because of broken disulfide bonds.

Pocket	Receptor	Ligand	Reason
IGF1R	5FXR	3NW7	No flexible residues
IGF1R	5FXR	3NW6	No flexible residues
MK10	4HYS	4L7F	No flexible residues
MK01	4GSB	5NHV	No flexible residues
MK10	1UKI	2G01	No flexible residues
SRC	3UQG	5D10	No flexible residues
JAK2	4F08	4D0W	No flexible residues
IGF1R	5FXR	1JQH	No flexible residues
MK10	1UKI	3RTP	No flexible residues
IGF1R	5FXR	2OJ9	No flexible residues
ACES	1JJB	1ACJ	No flexible residues
CDK2	3QQJ	3IG7	No flexible residues
JAK2	4F08	4E4M	No flexible residues
ACES	1JJB	1ZGB	No flexible residues
MK10	1UKI	3ELJ	No flexible residues
MK01	4GSB	4FV2	No flexible residues
MK01	4GSB	4ZZM	No flexible residues
IGF1R	5FXR	2ZM3	No flexible residues
MK01	4GSB	5LCJ	No flexible residues
SRC	3UQG	5J5S	No flexible residues
JAK2	4F08	5CF6	No flexible residues
IGF1R	5FXR	3LVP	No flexible residues
SRC	3UQG	3DQX	No flexible residues
ACES	1JJB	2CMF	No flexible residues
KIF11	4BXN	1X88	Broken spurious bond
KIF11	4BXN	2IEH	Broken spurious bond
KIF11	4BXN	2X7D	Broken spurious bond
KIF11	4BXN	3K3B	Broken spurious bond
CP2C9	1R9O	5W0C	Added spurious bond
FA10	1IQE	2RA0	Broken disulfide bond
FA10	1IQE	3KQB	Broken disulfide bond
FA10	2XBV	2FZZ	Broken disulfide bond
FA10	2XBV	2Y82	Broken disulfide bond

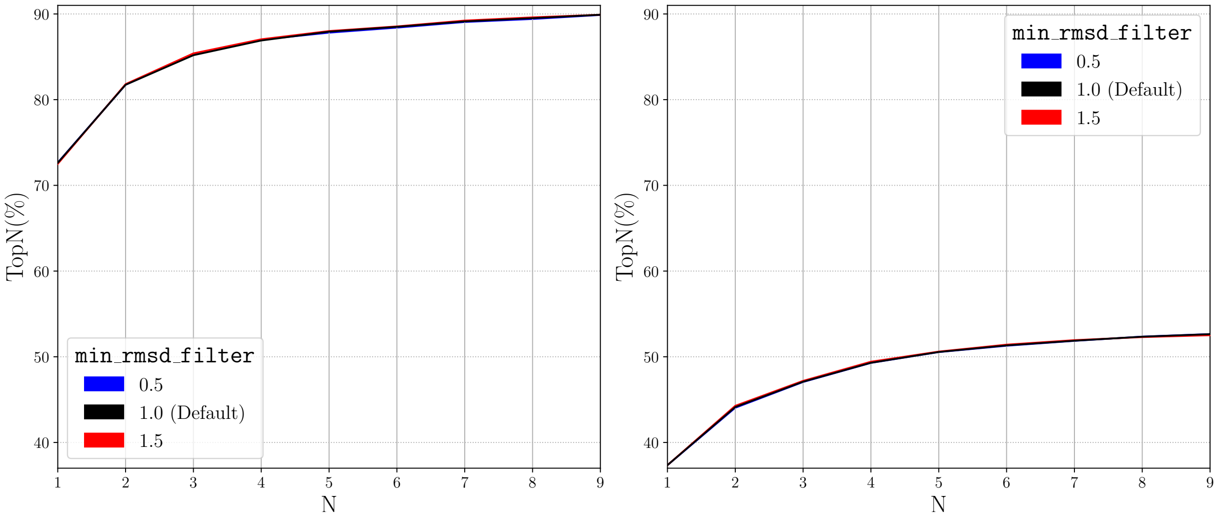

(a) Redocking (b) Crossdocking

Figure S9: Evaluating the effect on docking performance when the value of `min_rmsd_filter` is changed while using the Default Ensemble for rescoring on both the redocking and crossdocking datasets. TopN is the percentage of targets ranked above or at N with a RMSD less than 2Å

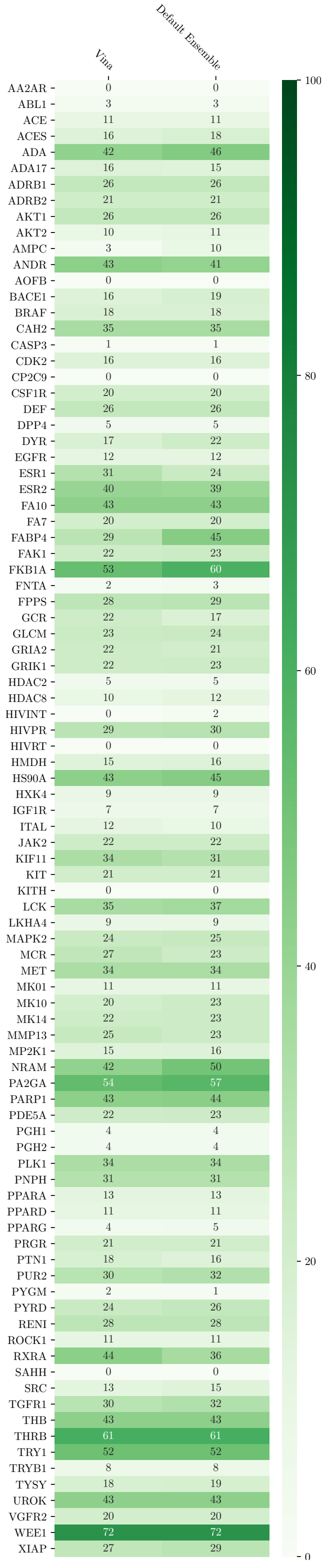

Figure S10: Cross-docking results using the whole protein as the defined binding pocket. Evaluating Top1(%) per pocket. Top1 is the percentage of top ranked targets with a RMSD less than 2Å.

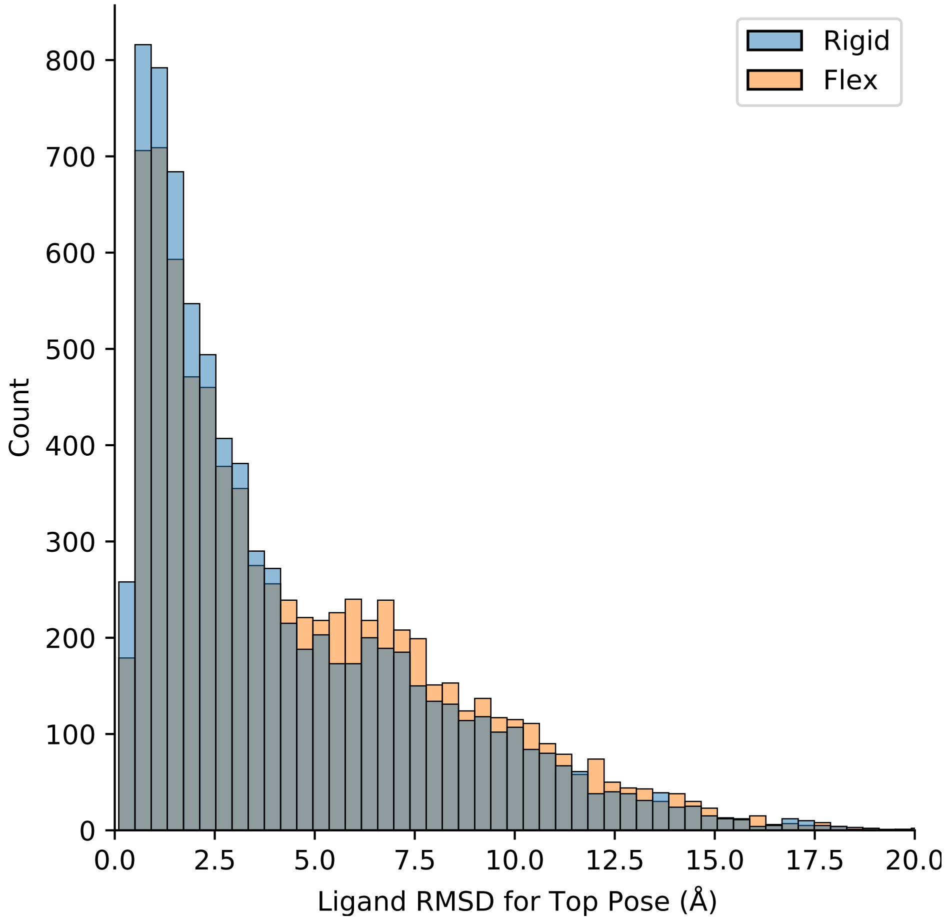

Figure S11: Ligand RMSD distributions for the top pose in the cross-docking dataset, for bot flexible and rigid docking.

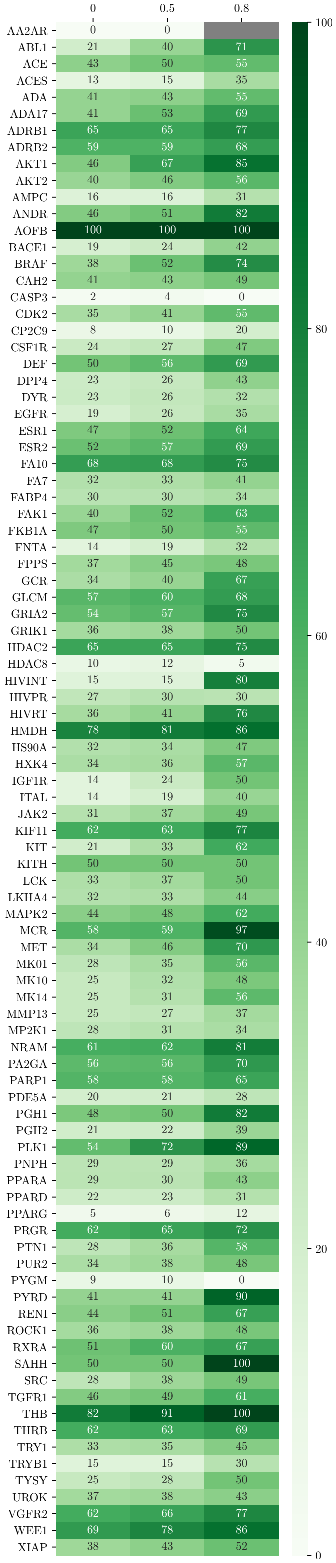

Figure S12: Thresholding the cross-docking results by CNNscore and evaluating Top1(%) per pocket. A pose is retained if the CNN outputs a score greater than the value indicated on the x-axis. Grey cells indicate that no poses are left in the pocket. Top1 is the percentage of top ranked targets with a RMSD less than 2Å.
